# 526. In Situ Colorimetric Nanofiber Membrane for Indicating Pathogen Invasion in Wounds

**DOI:** 10.1093/jbcr/irag033.223

**Published:** 2026-04-06

**Authors:** Farinaz Jonidi Shariatzadeh, Chelsea Luxen, Sarvesh Logsetty, Maurice Haff, Song Liu

**Affiliations:** University of Manitoba, Winnipeg, MB; ParaNano Wound Care LLC, Edmone, OK; University of Manitoba, Winnipeg, MB; ParaNano Wound Care LLC, Oklahoma City, OK; University of Manitoba, Winnipeg, MB

## Abstract

**Introduction:**

Infection in burn wounds remains a major clinical challenge that can delay healing and increase the risk of complications. Current methods for determining inhibitory bacterial presence in burn wounds are labor-intensive, time-consuming, and require specific training, often delaying intervention. Once physical symptoms appear, bacterial loads may already exceed critical thresholds, increasing systemic infection risk. To address this gap, we developed an in situ colorimetric nanofiber membrane that provides a visual indication of bacterial invasion at critical thresholds of presence. Unlike existing methods that require costly instrumentation, this colorimetric nanofiber membrane offers a cost-effective, easy to use solution for wound monitoring, changing color from yellow to green in response to bacterial activity. The membrane is compatible with commercially available foams and transparent films commonly used in burn wound care.

**Methods:**

A lipase-cleavable hemicyanine dye was synthesized and integrated into a nanofiber membrane through electrospinning of polyurethane and polyvinylpyrrolidone. The sensitivity and color-change response were evaluated against *Pseudomonas aeruginosa* and MRSA, both directly and in combination with commercial wound dressings, under conditions with and without artificial wound exudate. Cytotoxicity was assessed using an elution assay in accordance with ISO standards.

**Results:**

The colorimetric membrane demonstrated a detection limit of 1.0E+5 CFU/cm^2^, producing a distinct visual signal within 5 hours when in direct contact with agar. In the presence of foams without exudate, response occurred within 6 hours. Scanning electron microscopy confirmed the observed color change was triggered by bacterial activity. In the presence of wound exudate, increased fluid exposure enhanced lipase–dye interaction, accelerating the color change within 5 hours. Hydrophobic foams with a top barrier layer were incompatible, as they blocked enzyme–membrane interaction, whereas all transparent films were compatible and did not interfere with color visibility. However, turbid or tinted films reduced visual clarity. Cytocompatibility testing confirmed >75% viability in fibroblasts.

**Conclusions:**

This colorimetric nanofiber membrane enables continuous in situ monitoring for pathogen invasion at bacterial concentrations below the critical threshold of infection, while maintaining cytocompatibility.

**Applicability of Research to Practice:**

The visible color shift from yellow to green occurs without external instrumentation and can be visually observed in real-time without dressing removal, offering patients and clinicians a simple, low-cost burn wound monitoring tool to support timely intervention.

**Funding for the study:**

Natural Sciences and Engineering Research Council of Canada, the Canada Foundation for Innovation and Corporate Funding.

